# Varying ethics rules in clinical research and routine patient care – research ethics committee chairpersons’ views in Finland

**DOI:** 10.1186/1478-4505-12-15

**Published:** 2014-03-25

**Authors:** Elina Hemminki, Jorma I Virtanen, Piret Veerus

**Affiliations:** 1 National Institute for Health and Welfare, P.O. Box 30, 00271 Helsinki, Finland; 2 Hjelt Institute/Department of Public Health, University of Helsinki, P.O. Box 41, 00014 Helsinki, Finland; 3 Oulu University Hospital, P.O. Box 21, 90220 Oulu, Finland; 4 National Institute for Health Development, Hiiu 42, 11619 Tallinn, Estonia

**Keywords:** Clinical research, Cluster randomized trials, Emergency research, Ethics, Informed consent, Regulation

## Abstract

**Background:**

To present empirical data on how the variation in regulating clinical research and patient care was perceived in Finland between 2009 and 2012.

**Methods:**

Notes of interviews with 22 research ethics committee (REC) chairpersons were analyzed to identify whether differences in the regulation of clinical research and patient care were addressed. REC chairpersons’ opinions on three imaginary cases of clinical research projects challenging current research ethics rules (vignettes) were requested with a questionnaire; 18 of the 22 interviewed chairpersons responded.

**Results:**

Based on REC chairpersons’ interviews, the differences between care and research regulation were not considered important issues in Finland. In the vignettes, REC chairpersons’ assumptions on how their REC would decide varied in regard to allowing research without informed consent, while solutions that are not allowed by current law were even anticipated. Mostly, but not always, the chairpersons’ own personal view agreed with their REC.

**Conclusions:**

The distinction between care and research regulation has not been publicly challenged by Finnish RECs, even though it is a challenge when research relevant to health care is carried out. There is a need for debate and changes in laws and practices.

## Background

It is often argued that medical care aims to provide optimal care for an individual patient while research aims to produce generalizable knowledge for the benefit of future patients. However, this distinction is unclear in complex health care systems aiming for evidence-based care [1]. Faden et al. [2] have argued that classification schemes that bifurcate learning activities into two crude categories of research and practice are increasingly outmoded. The origin for this distinction has been traced to the influential 1979 Belmont report in the USA [3]. Before that, research and care were often intertwined [1].

Current problems in clinical research include the issue that some important questions have been neglected [4] and the applicability of research to real care situations is not clear [5]. To increase knowledge creation, it has been suggested that health care and research should be better integrated and the current norms distinguishing these two should be modified [4,6,7]. It has been suggested that research is a responsibility and a priority for both patients and health professionals [8-20], and that the integration of research within everyday clinical practice would help achieve a more efficient arrangement for research [11-16].

The purpose of this paper is to present empirical data on how the variation in regulating clinical research and patient care is perceived in Finland. We studied i) whether the distinction has been an issue in Finland (using expert interviews) and ii) how ethics committees are likely to decide about clinically relevant research projects challenging current research ethics rules, using three fictitious cases (vignettes).

### The context

Finland is one of the Nordic welfare states. Its population of about 5.4 million is homogenous and well-educated. Health service funding has two co-existing systems: a community based, tax-funded area-based system covering most in-patient care and most out-patient care, and private care in part subsidized by national health insurance. Some other sickness costs are also partly reimbursed. Finland has a strong tradition for clinical research [21]. All clinical research has to be reviewed by official local research ethics committees (RECs) or the central committee. The law defines two functions of the central committee: to handle multinational drug trials (since 2010 all drug trials) and to support local RECs in ethics principles and in organizing education. The support functions have been relatively modest and the composition of the committee is tailored for the review of drug trials. The committee can delegate the handling of drug trials to local RECs, and most have been delegated (Hemminki E, unpublished data 2014).

There is one binding international research ethics instrument, the Convention on Human Rights and Biomedicine (Oviedo Convention). Other international codes valid in Finland include the Declaration of Helsinki, the Universal Declaration on Bioethics and Human Rights, the International Ethical Guidelines for Biomedical Research Involving Human Subjects, the Good Clinical Practice Guidelines, and the additional protocol of the Oviedo Convention concerning Biomedical Research [22]. The codes start from a principle that research is an activity to be clearly separated from care and that these two have their own rules with different reasoning and responsibilities. The codes do not provide guidance on how the problems resulting from this division could be solved, nor do they state the course of action in cases where research blends with care or the situations in which informed consent can be exempted.

Different laws apply to care and research in Finland. The key law regulating medical research is the Medical Research Act, first passed in 1999 with amendments in 2004 and 2010. In 2004, the Clinical Trials Directive covering clinical trials on medicinal products in the EU was integrated into the Medical Research Act (as well as into drug legislation), defining drug trials as a subgroup of medical research with some special requirements. However, some principles from the EU directive were enlarged to cover medical research as a whole [23]. In all drug research, even in emergency situations, an informed consent is needed, either from patients themselves or their relatives or guardians; no waivers are allowed.

The main changes in 2010 were to enlarge the scope of the law from a narrow medical (physician led) perspective to a health research (including nursing research, studies with registers, and with human blood and tissue samples) perspective; to reduce the number of ethics committees from 21 to 5 (plus a central committee); to strengthen the role of the central committee; to allow children to have more rights to decide on research participation; and to administratively transfer the central committee to another institute (from the general medical ethics committee, which dealt with health and social care ethics, to a medico-legal body). The background document to change the Medical Research Act in 2009 was detailed, but there was no discussion of the relation between research and care.

The 2011 Health Care Act (Terveydenhuoltolaki) includes various statements on research and a statement on the need for health care to be based on evidence. However, no advice on how evidence-based health care is to be achieved is given and the law does not take a stand on differences in regulating care and research. The long background document to the law contains little on research and nothing on the integration of research and care.

## Methods

This current study is a part of a larger study, with data collected by various methods [21]. To answer our question on whether distinction between clinical research and patient care had been an issue, chair-persons of RECs were interviewed. To find out how ethics committees are likely to decide about clinically relevant research projects challenging current research ethics rules, we used three fictitious research projects (vignettes).

All chairpersons of the Finnish official medical RECs in continental Finland were contacted. Of the 25 chairpersons, 22 (18 men and 4 women) were interviewed in Finnish by one of two researchers, a dentist and a lawyer, in 2010–11. The chairpersons were approached as experts, meaning knowledgeable persons who were asked to describe their experience and views, rather than research subjects. That approach influenced how they were contacted and interviewed, as well as the style of vignettes. For the same reason, we decided not to use voice-recording. We thought that some experts may consider the topic sensitive and that “discussion style” would work better.

Interviews with REC chairpersons were semi-structured. An interview guide was made, but each interview was tailored for the interviewees’ position, expertise, and interests. The interviews lasted from 30 minutes to 2 hours. During the interviews, notes were made and a summary of the answers and discussion was written down after the interview. We did not have a specific question on the relation between care and research, but a number of related questions, such as: What is the main focus in regulating research? Is it clear which regulation should be used for which type of research? Which factors promote or hinder research? What are the possibilities for academic (non-commercial) research? What should be changed in research regulation?

All interview notes were read by one researcher (EH) and analyzed for the following themes: i) need for (clinical) research of health care, ii) optimality of current rules, iii) comparison of rules for care and research, and iv) any other aspect on the relation between care and research. If any of the four themes had been discussed, the relevant sections were transferred to a separate document. As the comments on research and care were few (see Results), there was no need to develop refined coding.

After the interview, the REC chairpersons were given a questionnaire presenting three fictitious cases (Additional file 1). The cases were designed to challenge current research ethics rules (all were planned to be made without informed consent), but were clinically relevant. They were modified and combined from actual research protocols. Chairpersons were first asked to select a response which most closely matched their opinions of how the study would be reviewed by their ethics committee. Secondly, chairpersons were asked whether they thought the review decisions would correspond with their own personal ethics. Additional open comments were asked with an ample empty space.

Most REC chairpersons completed the questionnaire during the interview, but due to time constraints some took it with them and sent their responses later via mail or e-mail. Of the 22 interviewed chairpersons, 18 (81%) returned responses; based on all 25 chairpersons initially approached, the response rate was 72%. Of the 25 approached, two declined to return the form and 5 did not send the form despite having originally indicated that they would. The answers in the fixed questions were tallied. Correspondence to chairpersons’ own ethical views were calculated by each answer alternative (Tables 1, 2, 3). Comments were counted and their content classified by the topic and the content (see Results).

**Table 1:** **Research ethics committee (REC) replies to a fictitious research protocol (Cluster randomized trial without informed consent (IC), case 1 in Additional file**1**): how they assumed their REC would decide and whether they agreed with the decision, numbers**^**1**^**(n of respondents = 18)**

	**REC decision**	**Own ethical view**		
		**Agreed**^**2**^	**Somewhat**^**2**^	**No**^**2**^
Accept no informed consent **(**IC)	4	4	0	0
IC for intervention from collective	3	2	1	0
IC for data collection from collective	2	1	1	0
IC from patients to collect clinical data	7	5	2	0
IC from patients to answer questionnaire	6	1	5	0
Other	0	–	–	–

^1^In REC decision REC chairperson could choose more than one option.

^2^Agreed: chairperson agreed fully; Somewhat: chairperson agreed somewhat; No: did not agree.

**Table 2:** **Research ethics committee (REC) replies to a fictitious research protocol (A study mixing care and research in intervention, case 2 in Additional file **1**): how they assumed their REC would decide and whether they agreed with the decision, numbers**^**1**^**(n of respondents = 18)**

	**REC decision**	**Own ethical view**		
		**Agreed**^**2**^	**Somewhat**^**2**^	**No**^**2**^
Accept no informed consent (IC)	8	5	3	0
IC for intervention from collective	3	3	0	0
IC for data collection from collective	1	0	0	1
IC from patients for intervention and data collection	8	5	3	0
Other	1	1	0	0

^1^In REC decisions REC chairperson could choose more than one option.

^2^Agreed: chairperson agreed fully; Somewhat: chairperson agreed somewhat; No: did not agree.

**Table 3:** **Research ethics committee (REC) replies to a fictitious research protocol (An emergency trial with a drug having sales license for another indication, case 3 in Additional file **1**): how they assumed their REC would decide and whether they agreed with the decision, numbers**^**1**^**(n of respondents = 18)**

	**REC decision**	**Own ethical view**		
		**Agreed**^**2**^	**Somewhat**^**2**^	**No**^**2**^
Accept no informed consent (IC)	0	–	–	–
IC for intervention from collective	4	0	4	0
IC for data collection from collective	3	1	2	0
IC afterwards	7	3	4	0
Other^3^	6	1	1	4

^1^In REC decision REC chairperson could choose more than one option.

^2^Agreed: chairperson agreed fully; Somewhat: chairperson agreed somewhat; No: did not agree.

^3^Other: includes rejections (3 chairpersons).

The whole project (MERGO Ethical review and administrative governance of clinical research) received a positive statement from the ethics committee of the National Institute for Health and Welfare (June 17, 2010, amendment Jan 27, 2011). All interviews were voluntary and interviewees knew the purpose.

## Results

### Interviews

We interviewed 22 ethics committee chairpersons. Variation in the rules between care and research was not explicitly brought up. However, some chairpersons in smaller hospitals said that research and clinical care are integrated and that this is as it should be. Some interviewees believed that the proposal to transfer the ethics handling of all medical research to the five university RECs decreased attractiveness of research in smaller hospitals. It was considered a problem, as research was considered useful to the hospital.

The researcher as the care-giving doctor was mentioned a few times, but it was mentioned as an ethical problem rather as a positive thing for integrating research and care. The concern was that voluntary participation may be endangered. In one interview, another view was given: researchers should not directly recruit patients, but it should occur via care-giving doctors.

### Ethics committee (REC) chairpersons’ views on three imaginary research projects

The first case was a cluster-randomized health service trial that mixed care and research and which did not seek informed consent from patients: 30 health centers were to be randomized in terms of receiving or not receiving a computer-aided decision-making tool for doctors; patients were to be asked their experiences afterwards by mailed anonymous questionnaires and patient outcomes were to be collected from patient records (Additional file 1).

The assumption of chairpersons on how their REC would decide about the project varied (Table 1). Four out of 18 chairpersons thought that their REC would accept the design without requiring informed consent from anyone. Three suggested that the informed consent could be asked from a collective, such as a municipality health board. Seven suggested that patients should be asked about collecting data from their routine clinical records and six would have required informed consent to be given even for the anonymous questionnaire.

Chairpersons’ own personal ethical view agreed with the assumed decision of their REC either fully or somewhat (Table 1). The requirement for informed consent in anonymous questionnaires was assumed to be a requirement of the REC more often (n = 6) than it was considered a requirement by the chairpersons themselves (only one fully agreed with this view). Four chairpersons had commented further on the fixed responses.

The second imaginary research project (vignette 2, Additional file 1) was an individually randomized health service trial that mixed care and research without informed consent from patients. Patients were to be randomized to a nurse or doctor as the first contact in an emergency room; both were existing practices in the study health centers but used unsystematically. The outcomes were health professionals’ opinions (collected by questionnaires) and patients’ health status (data collected from patient records). The researchers referred to the project as development work.

The assumptions of chairpersons on how they thought their REC would decide varied (Table 2). Eight out of 18 chairpersons anticipated that their REC would accept the design and eight anticipated that the REC would request informed consent from patients both for the intervention and the data collection, as well as to exclude patients who declined. Three anticipated informed consent from a collective for the intervention and one for the data collection (REC chairperson could choose more than one option).

The personal views of chairpersons on what was ethically correct were mostly in accord with what they assumed their REC would decide, but not fully (Table 2). In one case, the chairperson disagreed with the anticipated decision of their REC (informed consent for data collection from a collective); he thought that no informed consent was needed.

Five chairpersons had given additional comments. One wrote that, as the assignment to nurses and the assignment to doctors have both already been used, there is no ethical problem in randomizing this assignment. Four others defended their requirement for informed consent from patients. Three justifications were normative: interventions always require permission from the patient. Two said that randomization makes the difference. One chairperson provided an ethical argument: in a normal situation the receptionist would use a decision-making algorithm, while randomization would influence the care of an individual patient.

The third imaginary research project (vignette 3, Additional file 1) was an emergency trial with a licensed drug given by ambulance personnel without informed consent. No chairperson anticipated the study design being accepted (Table 3). Seven anticipated a requirement that informed consent be obtained afterwards. Some chairpersons (four chair-persons for the intervention and three for data collection) anticipated that their REC would suggest informed consent from a collective. Three chairpersons anticipated that their REC would give a negative statement for the study (reject).

In the case of vignette 3, REC chairpersons less often considered their own ethical valuations to be in agreement with the anticipated decisions of their REC than with the previous vignettes. Even explicit criticism of their REC decision was given. Of the three chairpersons who assumed that the project would be rejected, only one fully agreed with the REC decision and two thought that the decision was not ethically reasonable.

Vignette 3 also raised many additional comments and eight were on substance. Three chairpersons commented that all specified options in the vignette were against the current law, and two wrote that the current law was not good. One put it: “*The ethics committee should obey the law… Thus new evidence-based treatment will not be available*”. One wrote clearly that (the law) is wrong and should be changed. One suggested obtaining permission from a relevant authority (not a specified option), and three suggested asking for permission afterwards. One chairperson wrote that his REC would ask for permission afterwards either from the patient or a relative, but not in the case of dead patients. The comment implicitly made clear that the chairperson knew their practice (apparently also used in real situations) could be challenged on legal grounds, but he thought it was ethically correct.

## Discussion

The purpose of this paper was to present empirical data on how the variation in regulating clinical research and patient care has been perceived by Finnish experts, chairpersons of RECs. Our main finding was that this variation apparently had not been widely discussed in Finnish RECs, but the imaginary cases (vignettes) suggested the existence of a problem. In some other countries, variations in the ethics and regulations governing care and research have been well debated, but we found no empirical studies with which to compare our own results.

Based on the interviews with the chairpersons of the RECs, the distinction between regulations governing care and research has not been an important issue in Finnish practice or debates. The REC-chairpersons’ assumptions on how their REC were likely to decide suggested a large variation in the decision-making for research projects involving cluster randomized trials and those challenging current ethics rules. Quite likely, the presented studies would be allowed by some RECs but not by others. Thus, the decision would depend on the committee that the researcher had sent his application to. The personal ethics of most of the chairpersons agreed with the assumed decision of their REC, but not always. This, and the varying REC decisions, may suggest unfamiliarity with the issue, but also an active questioning of the current ethics rules.

The study includes a number of limitations. First, the decision on the imaginary study plans (vignettes) was based on the chairperson’s guess of his/her REC behavior. It is difficult to know how the decision would be in reality when a detailed study plan would be presented to a committee. The validity of the vignettes was not formally tested. However, the researchers within the group read and modified the vignettes to make them clear, and feed-back from the first chairperson interviews showed that vignettes were clear to the respondents.

Secondly, the interviewees were not voice-recorded and the quality of notes is likely to vary, e.g., how quickly and concisely the interviewee spoke and how much medical jargon he/she used. Voice-recording was considered, but rejected. We thought that “discussion style” would work better. We wanted to get their views and not the “official” ones stipulated by their position.

Chairpersons were not asked specifically about the issues of having different regulation for treatment and practice. However, the findings of chairperson interviews that the issue of distinction between regulations governing care and research has not been an important issue in Finland is consistent with other data. Our unpublished data from documents and interviews with other experts in research and health services other than research ethics committee chairpersons showed the same: the principle of having different rules for research and clinical care had not been challenged in public debate or publicly available documents, even though many considered clinical research important for health care. As the requirement for informed consent for all types of medical research is well known among the medical research community, proposals have been tailored from the beginning to suit the detailed requirements for informed consent.

However, our incidental observations from individual projects mixing care and research show that it has been an issue for individual researchers. We have observed that researchers have used definitional solutions to make research mixing care and research possible. Research projects have been defined as programs or care experiments to avoid unsuitable requirements entailed in research regulation. Projects have likewise been defined as non-medical research; such projects in Finland do not have binding ethics rules or legal requirements. It seems that some researchers are familiar with the problems relating to mixing care and research, but rather than raising the issue have chosen to search for *ad hoc* solutions. Possibly, these researchers have felt that changing the ethics codes and research law may be too challenging. It is also possible that the problems of permissions, quality assurance, and development work are known to some ethics committees and institutes overseeing health care in Finland, but related discussions do not occur in public.

The Finnish medical research law was recently (2010) changed, and that would have offered an opportunity to solve some of the problems, but the opportunity was not used. An explanation could be that there were other issues in research regulation and financing which dominated the interest of law makers and medical experts. A law on biobanks was drafted and in that context the use of existing data was debated. The 2010 research legislation proposed disbanding most official ethics committees and at the same time university hospitals were given increasing power to decide about the use of research money from the state. Smaller hospitals might have feared that they would lose research money. That could explain the research ethics committee chairpersons of smaller hospitals saying that research and clinical care are integrated and that this is as it should be.

The first vignette was a cluster randomized trial without informed consent. The requirements for informed consent in cluster randomized trials vary between countries, within a country, and by the type of trial [24]. The Finnish research legislation assumes an informed consent from the participants and does not advise on how to handle this kind of research. In this study, the concept of ‘a participant’ is not clear, as it was health care centers and not patients, that were randomized. Furthermore, patients are not expected to decide how physicians make their diagnosis. Many REC chairpersons chose the alternative of obtaining the information from a collective, which has been previously proposed for these types of studies [25], but is not recognized in the Finnish research law. If this would have been a real situation and a REC would not have accepted the proposed application, researchers might have defined the project as a development project, or the researchers would have modified the design to meet the REC requirements. In the last option, we argue that asking for patient consent would have weakened the study design and feasibility.

The main difference between the second and first vignettes was that patients, as opposed to health care units, were randomized. Often, the mere act of randomization is considered to define an activity to be research requiring patients’ consent, but this has also been challenged [2,24]. If the patients would have been asked for an informed consent to be randomized to a doctor or nurse, the study results would not have been useful in the organizing of an emergency care unit, as it would likely merely reflect patient preferences only.

The third vignette was an emergency trial with a drug already registered for another indication. This case explicitly made visible the fact that the legislation does not always agree with good ethics. The case was built in such a way that it was not possible to obtain informed consent from patients or their relatives, as would be required by the current law. All the formulated choices for the REC decisions were against the Finnish law, even though not against international codes or interpretations elsewhere of the EU clinical trials directive. The REC chairpersons’ answers to this case showed, besides a lack of knowledge of the current law by some chairpersons, a moral conflict between obeying the law and carrying out ethical decisions. In reality, researchers might have modified the plan to be a care experiment and used the drug as planned, but without concurrent controls. Doctors are free to use drugs for unlicensed purposes provided it is medically defendable. In some other European countries, informed consent can be waived in emergency drug trials [26,27], but not in Finland.

New research approaches are being increasingly used. They include epidemiological research using biobanks and registers, as well as trials testing care structures and care delivery (including randomized care). However, current research regulations and codes hinder these approaches [17]. Making a clear distinction between continuous improvement activities (including clinical effectiveness assessment and quality improvement) and public health programs is difficult. The current ethics rules are unsatisfactory for these approaches [2,4,6,25,28-31]. For example, the individual informed consent and right to withdraw from the study at all stages are poorly applicable in many of these studies. The concept of “learning health care”, which covers research and various other activities to obtain information to improve health care, has challenged the ethics rules [2].

This problem is most evident in research designs that cannot easily apply current requirements for informed consent and the right to withdraw [4,6]. Informed consent is the key practical tool to separate care and research [26]. In Finland, written informed consent is a mandatory requirement in medical research, but it is not sought in medical care, not even in cases involving extensive surgical operations. In medical care, an implicit permission is assumed.

The different standards applied to research and care have been discussed at least since the 1960s [1,2,11,18-20,32-34], but little has happened in terms of developing regulations. In recent years, discussion and activities have begun to emerge among policy makers in some countries [4,28,30,11,35]. In the UK, practices around medical research regulation have been streamlined to encourage research within and by health care [30,36]. A special registry that records a sub-class of randomized care – randomized health services studies – has been established as analogous to the registers of clinical trials [29].

Based on our study and on previous studies and observations, we recommend that, in Finland, the current laws and rules, or their interpretation, regulating research and its definitions should be revisited and modified to allow better integration of clinical research and ordinary health care. Particularly, informed consent should not be the sole method to avoid coercion and manipulation in research focusing in care delivery and community actions, and alternatives to individual informed consent should be searched for. Waivers for informed consent are needed. It is likely that these recommendations apply to many other European countries as well.

## Competing interests

The authors declare that they have no competing interests.

## Authors’ contributions

EH originated the idea, planned the analysis and prepared the draft manuscript. PV participated in the study design and commented on the manuscript. JV participated in the study design, collected data, and commented on the manuscript. All authors read and approved the final manuscript.

## Supplementary Material

Additional file 1 The questionnaire on fictitious cases (vignettes) presented to ethics committee chairpersons. Click here for file
