# Supplementary material for: Varying ethics rules in clinical research and routine patient care – research ethics committee chairpersons’ views in Finland
Source: Health Res Policy Syst. 2014 Mar 25;12:15. doi: 10.1186/1478-4505-12-15 (PMC3987656; doi:10.1186/1478-4505-12-15)
Supplement: Additional file 1 — The questionnaire on fictitious cases (vignettes) presented to ethics committee chairpersons. [file 1478-4505-12-15-S1.docx]

Additional file 1. The questionnaire on fictitious cases (vignettes) presented to ethics committee chairpersons

Introduction (to be modified by the interviewer): We would also like to ask your opinion about imaginary example cases. Would the following studies have been approved by your committee?

To those who were reminded by letter, the text was: Dear Chair-person. In addition to the interviews we would like to know which kind of stand your ethics committee would be likely to take in regard to the following imaginary example cases. We will handle your answers confidentially and the identity of individual responses will not be revealed in the publications. If you are no longer a chair-person, answer according to that what your former committee would have been likely to decide. On behalf of the MERGO project (name and signature of the responsible researcher EH).

Case 1 A cluster randomized trial without informed consent

Aim: To study how the use of computer-aided decision-making influences patients’ experiences and clinical outcomes.

Methods: Thirty health centers are randomized into the intervention and control centers. After 6 months, an anonymous questionnaire is sent to a random sample of patients visiting the center. The name lists are obtained from the centers, the questionnaires are sent by the center, but asked to be returned to the researchers. Clinical outcomes are collected by the researchers form the physicians’ notes, but not linked to the (anonymous) questionnaires. No informed consent is planned.

In your opinion, how would your ethics committee deal with this study in regard to informed consent (you may choose several):

1. Accept the current design without informed consent.

2. Require an informed consent for the intervention from a collective (e.g., Terveyslautakunta).

3. Require an informed consent for the clinical data collection from a collective.

4. Require a permission from each patient for researchers to collect clinical data.

5. Require a written informed consent in the (anonymous) questionnaire.

6. Other, please specify: ______________.

Would this decision also correspond with your personal view of what is ethically acceptable?

1. Fully

2. Somewhat

3. No

4. Other, please specify: _________________.

Comments

Case 2. A study mixing care and research (HSB)

Aim: Is it better that the first health professional in the health center emergency department is a doctor or a nurse. In the selected health centers both practices are used, but unsystematically.

Methods: Five health centers (päivystyspistettä), with the agreement of the medical and nursing staff, decide to randomize all patients entering the center into those to be first seen by a nurse and those to be seen by a doctor; randomization is done by a non-medical person in the reception. If the clinical situation requires, the first person can be changed at any time. Patients are not told that they are randomized, but a general description of the study and its reasons are given in the local newspapers. As outcome measures nurses’ and doctors opinions’ (measured by questionnaires) and patient outcomes are used, abstracted from records. All work is done as part of the health center normal work, and classified as development. However, a statement from your research ethics committee is asked for.

In your opinion, how would your ethics committee deal with this study in regard to informed consent (you may choose several):

1. Accept the current design without informed consent.

2. Require an informed consent for the intervention from a collective (e.g., Terveyslautakunta).

3. Require an informed consent for the data collection from a collective.

4. Require a change in the intervention so that each patient is asked for permission to be randomized and data collection from patient records (and excluding those who decline randomization).

5. Other, please specify: ______________.

Would this decision also correspond with your personal view of what is ethically acceptable?

1. Fully

2. Somewhat

3. No

4. Other, please specify: _________________.

Comments

Case 3. An emergency trial with drugs

Aim: To see whether an already registered (for another indication) drug prevents deaths among people having received a large scale brain infarct (stroke), if given immediately by ambulance personnel.

Methods: Ambulance personnel in all the ambulances of a city participate and randomize all eligible patients. The outcome (death or not) is recorded, as well as the surviving patients’ status on discharge from the hospital. The study is conducted by the care-taking doctors in the city hospital. Informing the patients (who are not capable of giving an informed consent at the time of the intervention) or their relatives following the intervention is not planned.

In your opinion, how would your ethics committee deal with this study in regard to informed consent (you may choose several):

1. Accept the current design without informed consent.

2. Require an informed consent for the intervention from a collective (e.g., Terveyslautakunta).

3. Require an informed consent for the clinical data collection from a collective.

4. Require afterwards a permission from each patient/relative (even from those who have died)for the intervention and to collect clinical data.

5. Other, please specify: ____________.

Would this decision also correspond with your personal view of what is ethically acceptable?

1. Fully

2. Somewhat

3. No

4. Other, please specify: _________________.

Comments
